# A Performance Evaluation of the Vitek^®^2 AST-N440 Card for Colistin Susceptibility Testing of Carbapenem-Resistant *Acinetobacter baumannii* Complex Isolates Using Broth Microdilution as the Reference Method

**DOI:** 10.3390/antibiotics15040404

**Published:** 2026-04-16

**Authors:** Dimitra Petropoulou, Anastasios Ioannidis, Christina Kaminioti, Christina Mparka, Evgenia Mitropoulou, Georgia Petropoulou, Polyxeni Karakosta, Georgios Alexandros Baziotis, Spyros Pournaras

**Affiliations:** 1 Laboratory of Microbiology, General Panarcadian Hospital of Tripolis Evangelistria, 22100 Tripolis, Greece; dimitra.petropoulou@gmail.com (D.P.); barka_ch@yahoo.gr (C.M.); eugkmitrop@gmail.com (E.M.); gogo.petrop@gmail.com (G.P.); 2 Laboratory of Clinical Microbiology, School of Medicine, Attikon University Hospital, National and Kapodistrian University of Athens, 12462 Athens, Greece; tasobi@med.uoa.gr (A.I.); ckaminioti@yahoo.gr (C.K.); p_karakosta@hotmail.com (P.K.); bazbaziotis@windowslive.com (G.A.B.)

**Keywords:** antimicrobial susceptibility testing, AST-N440, carbapenem-resistant *Acinetobacter baumannii*, colistin, CRAB, Vitek^®^2 AST-N440, diagnostic performance

## Abstract

**Background/Objectives:** Accurate determination of colistin (COL) in vitro activity against carbapenem-resistant *Acinetobacter baumannii* complex (CRAB) isolates remains challenging, as the reference broth microdilution (BMD) method is labor-intensive and not routinely implemented in most clinical laboratories. Semi-automated susceptibility methods for colistin in the clinical laboratory require validation. The present study evaluated the performance characteristics of the recently introduced Vitek^®^2 card AST-N440 for COL antimicrobial susceptibility testing (AST) on CRAB isolates compared with a BMD-based reference method (ComASP Colistin). **Methods:** A total of 176 single-patient CRAB isolates from two distinct tertiary Greek hospitals between 2024 and 2025 were included. COL susceptibility testing was performed using Vitek^®^2 AST-N440 and compared with BMD. Minimum inhibitory concentrations (MICs) were interpreted according to EUCAST breakpoints. Method performance was evaluated by calculating categorical (CA) and essential agreement (EA), sensitivity, specificity, positive and negative predictive values (PPV/NPV), and major (ME) and very major error rates (VME) according to ISO 20776-2. **Results:** Compared with BMD, AST-N440 showed a sensitivity of 89.6% and a specificity of 62.3%, with a PPV and NPV of 81.7% and 76.0%, respectively. The CA (80.1%) and the EA (46.0%) were below ISO acceptance criteria. The VME rate was 10.4%, and the ME rate 37.7%. Identical MIC values were observed in 25.0% of the isolates, while Vitek^®^2 reported lower and higher MIC values than BMD in 46.6% and 28.4% of isolates, respectively. **Conclusions:** The Vitek^®^2 AST-N440 card performed suboptimally for COL susceptibility testing in CRAB isolates. Further validation of automated systems for COL AST is needed.

## 1. Introduction

Carbapenem-resistant *Acinetobacter baumannii* (CRAB) has been designated a Priority 1 (critical) pathogen by the World Health Organization (WHO) in its global priority pathogens list [1,2,3]. In Europe, CRAB rates vary widely, with particularly high prevalence in Southern and Eastern regions, where resistance rates may exceed 50% [4]. In Greece, invasive CRAB isolates have consistently exceeded 94% since 2017 [5,6]. Due to its persistence in the healthcare environment and its ability to acquire high levels of antibiotic resistance, *Acinetobacter* spp. infections represent a major therapeutic challenge for hospitalized patients [7].

Regardless of the available treatment recommendations from the Infectious Diseases Society of America (IDSA) and the European Society for Clinical Microbiology and Infection (ESCMID), observational evidence indicates that current treatment options are of limited efficacy [8,9]. Polymyxins, along with tigecycline, ampicillin/sulbactam and cefiderocol, are the preferred treatment options as monotherapy or as the main agents of a combination therapy. Colistin (COL) retains significant clinical effectiveness but it is difficult to dose correctly and shows significant side effects [10,11,12]. To avoid inappropriate COL administration, accurate in vitro antimicrobial susceptibility testing (AST) is highly recommended; however, the available assays confer challenges for the clinical laboratories. Broth microdilution (BMD) is the reference method for COL AST, suggested by both EUCAST (The European Committee on Antimicrobial Susceptibility Testing) and the CLSI (Clinical & Laboratory Standards Institute) [10,13]. Given the increasing reliance on COL for the management of CRAB infections and the technical limitations associated with its susceptibility testing, independent evaluations of newly introduced automated AST panels are essential. So far, studies have shown that semi-automated systems that include COL AST have failed to perform with the same effectiveness. New cards for COL AST have been introduced in recent years, incorporating a reformulation of COL aimed at improving analytical performance [14]. EUCAST has stated that it does not systematically evaluate semi-automated commercial systems such as Vitek^®^2, highlighting the need for independent performance assessments against the reference BMD method [15]. To date, limited data are available regarding the performance of the updated Vitek^®^2 AST-N440 cards. To the best of our knowledge, only one study has specifically evaluated the performance of the new AST-N439 card in *A. baumannii*, reporting suboptimal agreement with BMD and highlighting concerns regarding reliable detection of COL resistance [16]. To address this issue, the present study aimed to assess COL susceptibility testing performed by Vitek^®^2 using the recently introduced Vitek^®^2 AST-N440 card, which incorporates a reformulated COL preparation, compared with the reference BMD method, in a collection of 176 CRAB isolates from Greece. Given the limited availability of independent data evaluating the performance of this updated card, particularly in high-resistance settings, this study seeks to contribute new evidence regarding its reliability in routine clinical microbiology laboratories.

## 2. Results

All isolates were resistant to carbapenems, with imipenem (IMP) and meropenem (MER) MICs ≥ 8 mg/L. Resistance rates to antimicrobials were 100% for IMP, MER, imipenem/relebactam (IMR), and amikacin (AMK). The MIC_50/90_ values of the comparator antimicrobials were IMP (>8/>8), MER (>8/>8), IMR (>8/>8), and AMK (>32/>32) mg/L, respectively. The BMD and Vitek^®^2 AST-N440 MIC_50/90_ values for COL were 8/>16 and 4/>4 mg/L, respectively (Table 1).

Using the reference BMD method, 115/176 isolates (65.3%) were classified as COL-resistant. Of concern, a higher number of isolates (126 out of 176, 71.6%) were resistant to COL by Vitek^®^2 AST card N440 (Table 1).

The analytical characteristics of AST-N440 showed a high sensitivity of 89.6% compared with the reference BMD (Table 1). The specificity, NPV and PPV of Vitek^®^2 AST card N440 were relatively lower (62.3%, 76.0% and 81.7%, respectively) (Table 1).

**Figure 1:**
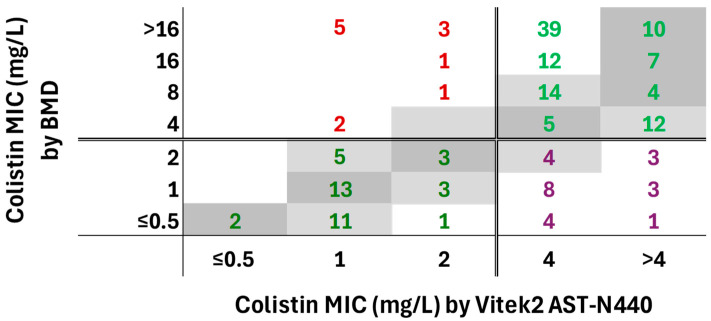
Cross-tabulation of colistin MICs obtained by Vitek^®^2 AST-N440 and BMD for 176 CRAB isolates. Dark gray cells represent identical MIC values, and light gray cells indicate MICs within ±1 doubling dilution. Double lines denote EUCAST breakpoints (2 mg/L). Green bold numbers indicate correct categorical classification, purple bold numbers indicate major errors, and red bold numbers indicate very major errors.

When compared with BMD, AST-N440 showed a CA of 80.1% and an EA of 46.0%, not satisfying the ISO acceptance criteria (≥90%). The VME rate was 10.4% (12/115 BMD-R isolates), and the ME rate was 37.7% (23/61 BMD-S isolates), exceeding ISO-defined acceptable limits (Table 2, Figure 1).

MIC distributions obtained by BMD and Vitek^®^2 are shown in Table 3. Among the 126 isolates with MIC ≥ 4 mg/L by Vitek^®^2, 103 also had MIC ≥ 4 mg/L by BMD.

Identical MIC values were observed in 44/176 isolates (25.0%). Vitek^®^2 reported lower MIC values than BMD in 82/176 isolates (46.6%) and higher MIC values in 50/176 (28.4%). Among the discrepant isolates, +1 and +2 doubling dilution differences were observed in 30 (22.7%) and 12 (9.1%) isolates, respectively, while ≥+3 dilution differences occurred in eight isolates (6.1%). Differences of −1 dilution were observed in 19 (14.4%) isolates, whereas ≤−2 dilution differences were observed in 63 isolates (47.7%) (Figure 1).

Spearman’s rank correlation demonstrated a weak but statistically significant positive correlation between Vitek^®^2 and BMD MIC values (ρ = 0.346, *p* < 0.001). Bland–Altman analysis of log2-transformed MIC values demonstrated a mean difference of −0.88 ± 1.97 dilutions, between Vitek^®^2 and BMD, with 95% limits of agreement ranging from −4.74 to 2.99 dilutions. Cohen’s kappa coefficient for CA between methods was 0.542, (*p* < 0.001). Stratified analysis according to BMD MIC ranges showed that discrepancies between methods were more frequently observed among isolates with MIC values close to the susceptibility breakpoint (2 mg/L) (46.7%) compared with isolates with MIC values ≤ 1 mg/L (34.8%) and ≥4 mg/L (10.4%).

## 3. Discussion

The increasing use of polymyxins as last-line therapeutic options has underlined the need for accurate and easy-to-implement COL AST in routine clinical laboratories. Automated and semi-automated systems such as Vitek^®^2 are widely used due to their ease of use, rapid turnaround time and integration into routine workflows. However, reliable detection of COL resistance remains challenging, and independent validation of newly introduced testing cards is essential. In this context, the present study evaluated the performance of the Vitek^®^2 AST-N440 card, which incorporates a reformulated COL preparation intended to improve analytical performance compared with earlier Vitek^®^2 COL cards, using BMD as the reference method, in a collection of CRAB isolates. Our findings demonstrated suboptimal performance of the automated system according to the ISO 20776-2 criteria [17]. The EA did not reach the acceptable threshold, the CA remained below 90%, and a high ME rate was observed despite good sensitivity for resistance detection. Only 25% of isolates had identical MICs, while a substantial proportion of isolates exhibited ≥2 doubling dilution differences. The correlation between Vitek^®^2 and BMD MICs was weak but significant, reinforcing quantitative discordance between methods.

COL AST has been extensively investigated over the last decade due to well-recognized methodological difficulties. International working groups have discouraged the use of diffusion-based methods and recognize BMD as the only valid reference method, while simultaneously emphasizing the need for validation of automated AST systems [10,13,15,18]. Earlier studies evaluating previous Vitek^®^2 COL cards (cs01n) consistently reported heterogeneous and often problematic performance. Of concern, investigations for *A. baumannii* documented high rates of VMEs, variable CA and poor EA. Large studies using earlier cards reported relatively high CA rates [19,20,21,22], heterogenous results concerning EA rates, ranging between 55% and 88.9% [19,20,22,23,24], and VME rates ranging from approximately 30% to 50% [20,22,23,24]. Mes, collectively, were reported low [6,20,22,24]. Several authors concluded that Vitek^®^2 could not replace BMD for COL susceptibility testing. Importantly, inaccurate results were particularly common for isolates with MIC values close to the susceptibility breakpoint, emphasizing the difficulty of testing COL reliably [24]. To address these limitations, Vitek^®^2 cards incorporating a reformulated COL preparation (cs02n, including AST-N439 and N440) were introduced [14]. However, independent data evaluating cards (e.g., AST-N439, N440) incorporating the new COL formulation (cs02n) remain scarce. Manufacturer-reported data have suggested high agreement with BMD, with EA values approaching 98% [14]. In contrast, the only independent study available to date reported that its performance did not meet ISO acceptance criteria in *A. baumannii* [16]. That study demonstrated an overall CA and EA lower than 90% across Gram-negative isolates, and a CA of 77.9% and an EA of 83.7% across *A. baumannii* isolates, highlighting a consistent tendency of Vitek^®^2 to underestimate COL MIC values compared with BMD.

Our findings are consistent with and expand upon the observations of Kim et al. [16]. In their multicenter study of carbapenem-non-susceptible Gram-negative bacilli, Vitek^®^2 demonstrated a CA and EA of 77.9% and 83.7%, respectively, for *A. baumannii* isolates, with both below ISO 20776-2 acceptance criteria. In accordance with Kim et al. [16], in our CRAB-only cohort, CA (80.1%) and EA (46.0%) were clearly below acceptable limits, with EA being markedly lower than that reported by the authors. The low EA observed in the present study appears to reflect both methodological considerations and true quantitative discrepancies between methods. Importantly, a substantial proportion of isolates exhibited MIC differences greater than one doubling dilution between Vitek^®^2 and BMD, indicating true quantitative discordance rather than solely an analytical issue. Differences may partly reflect the narrower MIC range of the Vitek^®^2 system compared with the broader dilution range of the reference BMD method. In addition, biological factors such as heteroresistance in *A. baumannii* may further contribute to variability in MIC determination and reduced EA between methods [25]. Furthermore, Kim et al. could not assess VME and ME due to the absence of a susceptible category for COL under CLSI criteria [16]. In contrast, we were able to quantify clinically relevant error rates under EUCAST criteria, highlighting the risk of false-susceptible and false-resistant results in routine testing. Notably, we observed a VME rate of 10.4%, substantially exceeding the ISO acceptable threshold (≤1.5%), alongside a high ME rate (37.7%) and limited specificity (62.3%), despite relatively good sensitivity for resistance detection (89.6%). VMEs were predominantly associated with underestimation of COL MIC values by Vitek^®^2 compared with BMD, particularly for isolates in which the reference MIC values were several doubling dilutions above the susceptibility breakpoint. In contrast, MEs were mainly observed near the breakpoint, where small quantitative differences resulted in categorical misclassification and contributed to the relatively low specificity observed for the automated system. The elevated error rates observed in this study may reflect methodological and biological factors affecting COL susceptibility testing. These include the physicochemical properties of COL, the limited dilution range of the Vitek^®^2 AST-N440 card, and variability in MIC determination near the susceptibility breakpoint, potentially influenced by heteroresistant subpopulations. VMEs represent false-susceptible results and are considered the most clinically critical error type, as they may lead to inappropriate administration of COL therapy in truly resistant isolates. This risk is particularly relevant in the context of CRAB infections, where therapeutic options are limited and treatment decisions often rely on accurate susceptibility data. MEs represent false-resistant results and may result in withholding the use of COL in cases where it may remain an effective therapeutic option. Given the narrow therapeutic window and toxicity of COL, reliable MIC determination is essential.

In a setting such as CRAB infections, where therapeutic options are already limited, this level of false susceptibility is concerning. The weak but statistically significant correlation between MIC values obtained by Vitek^®^2 and BMD (ρ = 0.346) further supports the presence of quantitative discrepancies between methods. Bland–Altman analysis demonstrated a tendency of Vitek^®^2 to report lower MIC values compared with BMD and wide limits of agreement, while Cohen’s kappa indicated moderate CA. Stratified analysis further showed that discrepancies were most frequent among isolates with MIC values at the susceptibility breakpoint, which may lead to categorical misclassification and influence clinical interpretation of susceptibility results. Analysis of doubling dilution differences demonstrated that a considerable proportion of isolates exhibited ≥2 dilution differences, reinforcing the lack of strong quantitative agreement. Overall, the AST-N440 card demonstrated suboptimal performance according to ISO 20776-2 acceptance criteria [17]. Collectively, these findings suggest that MIC values generated by Vitek^®^2 should be interpreted with caution and should not be used for quantitative MIC-guided optimization of COL dosing. Despite technical modifications, challenges in automated COL susceptibility testing persist.

The present study has several notable strengths. First, a substantial number of CRAB isolates were included, providing a sufficient dataset for the performance evaluation of the Vitek^®^2 AST-N440 card. Second, this study contributes to the limited independent evidence regarding the performance of the new Vitek^®^2 COL card and provides additional data on its ability to detect COL resistance and estimate VME rates. This study also has limitations. MIC determination by visual inspection of BMD plates may introduce minor observer variability, although standardized procedures were followed. In addition, the study included only CRAB isolates, and therefore the findings cannot be generalized to non-CRAB *A. baumannii* or to other Gram-negative pathogens, although the vast majority of *A. baumannii* isolates in Greece are CRAB. Moreover, the study population consisted predominantly of COL-resistant isolates (65.3%), reflecting the high prevalence of resistance in Greece. This distribution may have influenced predictive values, particularly the PPV and NPV, which depend on resistance prevalence. In settings with a lower prevalence of COL resistance, the NPV of the test would be expected to increase, while the PPV may decrease. Finally, molecular characterization of COL resistance mechanisms, including chromosomal alterations and plasmid-mediated determinants, was beyond the scope of the present study and was not performed. Such analyses could have provided additional insight into the biological basis of discrepant results between Vitek^®^2 and BMD. Similarly, evaluation of heteroresistance was not performed and should be addressed in future studies combining phenotypic and molecular approaches [25].

In conclusion, the present study provides independent evidence that the updated Vitek^®^2 AST-N440 card does not meet current performance criteria for COL susceptibility testing in CRAB isolates and further validation by BMD is needed.

## 4. Materials and Methods

The study included 176 non-duplicate CRAB isolates recovered from two Greek clinical laboratories from March 2024 through December 2025; isolates were selected randomly during the study period and were not consecutive. The clinical samples included blood, bronchial secretions, urine, superficial or deep tissue wounds, peritoneal effusions and intra-abdominal secretions. Initial bacterial identification was performed using Vitek^®^2 compact system (bioMérieux, Marcy-l’Étoile, France). All isolates were characterized and confirmed to be *A. baumannii* by matrix-assisted laser desorption/ionization time-of-flight mass spectrometry (Bruker Daltonik GmbH, Bremen, Germany). Carbapenem resistance and susceptibility testing of antimicrobials with potential activity against CRAB was initially determined by routine AST using the Vitek^®^2 system. Minimum inhibitory concentrations (MICs) were interpreted according to EUCAST guidelines and breakpoints (version 16.0), where applicable [10].

COL susceptibility testing was performed for all isolates using the Vitek^®^2 AST-N440 according to the manufacturer’s instructions. The analytical MIC range for COL on the Vitek^®^2 AST-N440 card was ≤0.5 to ≥4 mg/L in two-fold dilutions. Reference MIC values for COL were determined using a BMD-based commercial kit ComASP^®^ Colistin (Liofilchem^®^, Roseto degli Abruzzi, Italy), in cation-adjusted Mueller–Hinton broth (CAMHB) following EUCAST guidelines [10,18]. The commercial BMD-based system used in this study follows the standardized BMD methodology recommended by EUCAST and is widely used in routine clinical microbiology laboratories, providing standardized reagents and procedures for colistin susceptibility testing.

The BMD MIC test range was 0.25 to 16 mg/L. COL susceptibility was interpreted according to EUCAST breakpoints (Susceptible (S) ≤ 2 mg/L; Resistant (R) > 2 mg/L) [10]. Quality control was performed using *Escherichia coli* ATCC 25922 and *Pseudomonas aeruginosa* ATCC 27853. All control MIC values were within EUCAST acceptable ranges; MICs for ATCC 25922 and ATCC 27853 were 0.25 mg/L and 0.5 mg/L, respectively [10]. BMD plates were incubated at 35 °C for 18–20 h, and MIC endpoints were determined by visual inspection as the lowest concentration inhibiting visible growth.

The performance of the Vitek^®^2 AST-N440 card was evaluated using BMD as the reference method. Sensitivity, specificity, positive predictive value (PPV), and negative predictive value (NPV) for detection of COL resistance were calculated.

Agreement was assessed based on a comparison of category (qualitative) and MIC (quantitative). Categorical agreement (CA) represented the proportion of isolates grouped in the same interpretive category (S or R) by Vitek^®^2 compared with BMD, and essential agreement (EA) was defined as the proportion of isolates that differed by one doubling dilution by Vitek^®^2 compared with BMD, according to ISO 20776-2:2021 [17]. To allow comparison of EA and to evaluate the impact of off-scale MIC values, MIC values of >4 mg/L for Vitek^®^2 were considered to have EA with BMD values of >4 mg/L (i.e., 8, 16 and ≥32 mg/L), but not with BMD values of 4 mg/L, for which exact quantitative agreement could not be assumed. Very major errors (VMEs) were defined as isolates categorized as susceptible to COL by Vitek^®^2 but resistant by BMD (false-susceptible results); major errors (MEs) were defined as isolates categorized as resistant by Vitek^®^2, but susceptible by BMD (false-resistant results). Acceptable performance criteria were defined according to ISO 20776-2 as: EA ≥ 90%, CA ≥ 90%, VME ≤ 1.5%, and ME ≤ 3% [17]. Minor errors were not evaluated because the EUCAST guidelines do not include an intermediate susceptibility category [10].

Spearman’s rank correlation coefficient was calculated to assess correlation between Vitek^®^2 and BMD MIC values. Agreement between methods was further evaluated using Bland–Altman analysis of log2-transformed MIC values and Cohen’s kappa coefficient. Stratified analysis according to BMD MIC ranges (≤1 mg/L, 2 mg/L, and ≥4 mg/L) was performed to examine the distribution of discrepancies across clinically relevant MIC categories. Statistical analysis was performed using Microsoft Excel (Microsoft Corp., Redmond, WA, USA) and IBM SPSS Statistics (version 30.0, IBM Corp., Armonk, NY, USA). A *p*-value < 0.05 was considered statistically significant.

## Figures and Tables

**Table 1:** Colistin susceptibility rates, MIC_50_, MIC_90_ and the performance characteristics of BMD and AST Vitek^®^2 card N440 on 176 CRAB isolates.

AST Method	Susceptible (Rate), %	Resistant (Rate), %	MIC_50_	MIC_90_	Sensitivity (%)	Specificity (%)	PPV (%)	NPV (%)
BMD	(61/176), 34.7	(115/176), 65.3	8	>16				
Vitek^®^2 AST-N440	(50/176), 28.4	(126/176), 71.6	4	>4	89.6	62.3	81.7	76.0

AST, antimicrobial susceptibility testing; BMD, broth microdilution; NPV, negative predictive value; PPV, positive predictive value.

**Table 2:** Colistin susceptibility rates, EA (essential agreement), CA (categorical agreement) and errors of BMD and AST Vitek^®^2 card N440 on 176 CRAB isolates.

AST Method	Susceptible (Rate), %	Resistant (Rate), %	EA (Rate), %	CA (Rate), %	VMEs (Rate), %	MEs (Rate), %	Spearman’s Coefficient
BMD	(61/176), 34.7	(115/176), 65.3	-	-	-	-	ρ = 0.346 (*p* < 0.001)
Vitek^®^2 AST-N440	(50/176), 28.4	(126/176), 71.6	(81/176), 46.0	(141/176), 80.1	(12/115), 10.4	(23/61), 37.7	

AST: antimicrobial susceptibility testing; BMD: broth microdilution; CA: categorical agreement; EA: essential agreement; MEs: major errors; VMEs: Very major errors.

**Table 3:** Colistin MIC (mg/L) distribution by method of 176 isolates.

AST Method	Number of Isolates per MIC						
	≤0.5	1	2	4	8	16	>16
BMD	19	27	15	19	19	20	57
Vitek^®^2 AST-N440	2	36	12	86	(>4) 40 ^©^	-	-

AST: antimicrobial susceptibility testing; BMD: broth microdilution. ^©^ For the purpose of essential agreement calculation, MIC values reported as >4 mg/L by Vitek^®^2 were considered to have essential agreement only with BMD values > 4 mg/L (i.e., 8, 16, or ≥32 mg/L), but not with BMD values of 4 mg/L.

## Data Availability

Data from this study can be available on request.

## Abbreviations

The following abbreviations are used in this manuscript:

AMK	Amikacin
AST	Antimicrobial susceptibility testing
BMD	Broth microdilution
CA	Categorical agreement
CLSI	Clinical & Laboratory Standards Institute
COL	Colistin
CRAB	Carbapenem-resistant *A. baumannii*
EA	Essential agreement
ECDC	European Centre for Disease Prevention and Control
ESCMID	European Society for Clinical Microbiology and Infection
EUCAST	The European Committee on Antimicrobial Susceptibility Testing
IDSA	Infectious Diseases Society of America
IMP	Imipenem
IMR	Imipenem/relebactam
ISO	International Organization for Standardization
MALDI-TOF	Matrix-assisted laser desorption/ionization time-of-flight analyzer
ME	Major error
MER	Meropenem
MIC	Minimal Inhibitory Concentration
NPV	Negative predictive value
PPV	Positive predictive value
VME	Very major error
WHO	World Health Organization
